# Developing a novel rabbit model of atherosclerotic plaque rupture and thrombosis by cold-induced endothelial injury

**DOI:** 10.1186/1423-0127-16-39

**Published:** 2009-04-04

**Authors:** Shun-Miao Fang, Qing-Hua Zhang, Zhi-Xin Jiang

**Affiliations:** 1 Department of Cardiology, Southern Medical University (SMF), Guangzhou, 510515, China; 2 Experimental Centre, the 305 Hospital of Chinese People's Liberation Army (QHZ, ZXJ), Beijing, 100017, PR China

## Abstract

**Background:**

It is widely believed that atherosclerotic plaque rupture and subsequent thrombosis leads to acute coronary events and stroke. However, study of the mechanism and treatment of human plaque rupture is hampered by lack of a suitable animal model. Our aim was to develop a novel animal model of atherosclerotic plaque rupture to facilitate the study of human plaque disruption and thrombosis.

**Methods:**

28 healthy male New Zealand white rabbits were randomly divided into two groups: rabbits in group A (n = 12) were only fed a high-fat diet for eight weeks; rabbits in group B (n = 16) underwent cold-induced endothelial injury with liquid nitrogen, then were given a high-fat diet for eight weeks. After completion of the preparatory regimen, triggering of plaque rupture was attempted by local injection of liquid nitrogen in both groups.

**Results:**

All rabbits in group B had disrupted plaques or rupture-driven occlusive thrombus formation, but none in group A showed any effects. More importantly, the cold-induced plaques in our model were reminiscent of human atherosclerotic plaques in terms of architecture, cellular composition, growth characteristics, and patterns of lipid accumulation.

**Conclusion:**

We successfully developed a novel rabbit model of atherosclerotic plaque rupture and thrombosis, which is simple, fast, inexpensive, and reproducible, and has a low mortality and a high yield of triggering. This model will allow us to better understand the mechanism of human plaque rupture and also to develop plaque-stabilizing therapies.

## Background

It is now recognized that atherosclerotic plaque rupture and ensuing occlusive thrombus formation play an important role in the onset of two major causes of death in developed countries: acute coronary syndrome (ACS) and ischemic stroke[1,2]. By its very nature, plaque rupture is difficult to study directly in humans. A good animal model would help us to understand not only how plaque rupture and thrombus formation occur, but also how to take measures to prevent these from happening.

Over the years, many animal models of atherosclerosis have been developed. In recent years, several models of plaque rupture and thrombosis have also begun to emerge. However, all of the existing models, such as balloon-induced and biological or mechanical triggering rabbit models [3-6], the Watanabe heritable hyperlipidemic (WHHL) rabbit model [7], and the apolipoprotein E (ApoE) or the LDL-receptor mice models [8-12], suffer the drawback that they lack direct evidence of characteristic plaque rupture accompanied by platelet and fibrin-rich occlusive thrombus at the rupture site [13]. There are additional disadvantages of the existing models, such as the long preparatory period, the complicated manipulation, the high cost of development, the low yield of triggering, and the high mortality, which hamper ensuing large-scale studies.

In this study, we aimed to develop a novel rabbit model of atherosclerotic plaque that would be associated with true plaque rupture and also a rupture-driven occlusive thrombus formation that is structurally similar to that found in humans [1]. More importantly, the advantages of our model over other currently used animal models are that it is simpler, faster, less expensive, and more reproducible, and has a lower mortality and higher yield of triggering.

## Methods

### Animal preparation

28 healthy male New Zealand white rabbits (about 3-month-old) were randomly divided into two groups: rabbits in group A (n = 12) were fed a high-fat diet only (containing 1% cholesterol, 3% lard, and 15% yolk) for 8 weeks. In contrast, rabbits in group B (n = 16) first underwent cold-induced endothelial injury with liquid nitrogen, then were fed the same high-fat diet as group A for 8 weeks. Cold-induced endothelial injury was performed by injection of liquid nitrogen through a carotid artery. The rabbits were anesthetized with an intravenous injection of pentobarbital sodium (30 mg/kg). After the surgery, the animals were allowed to recover and conventional antibiotics were required to prevent infection. All rabbits survived until the time of attempted triggering, with the exception of one in group B that was killed at random in order to observe the severity of endothelial injury by Evans blue staining. Three rabbits in group B were also taken at random to ensure the establishment of atherosclerotic plaque, and the remaining rabbits in two groups were triggered by liquid nitrogen. All procedures that used animals were conducted in compliance with the administrative act for experimental animals in China.

### Cold-induced endothelial injury by liquid nitrogen

To induce atherosclerotic lesions, liquid nitrogen was injected into the right carotid artery of each rabbit in group B. With the rabbit under sufficient anesthesia, a midline incision was made on the neck. The right carotid artery was surgically exposed, and a 4-cm segment of artery was isolated by two artery clamps. A 1-ml aseptic syringe needle was immediately inserted into the proximal end of the segment. Blood was rinsed from the segment with phosphate-buffered saline, and the segment was evacuated completely, then liquid nitrogen (about 0.5 ml) was injected as quickly as possible into the empty artery through another 1-ml syringe via the indwelling needle. The cold liquid nitrogen gasified instantly in the artery. To ensure endothelial injury of the carotid artery, this process was repeated three times in each rabbit over a period of about two minutes. The segment was then rinsed with phosphate-buffered saline, the needle was withdrawn, and the artery clamps were loosened from the distal end to the proximal end. Circulation was re-established, hemostasis was ensured, and the surgical incision was closed. To evaluate the severity of endothelial injury by liquid nitrogen, 24 hour after surgery, one rabbit in group B was taken at random to receive an intravenous injection of 0.5% Evans blue dye (2 ml/kg). Two hours later, the animal was killed, the carotid arteries on both sides were dissected and excised, and the intimal surfaces were exposed by an anterior longitudinal incision of the vessel.

### Triggering by liquid nitrogen

To induce the triggering of plaque rupture in both groups, 8 weeks later, liquid nitrogen was injected into the right carotid aortic segment with a 1-ml syringe. The triggering was performed at the same injection site and according to the method of cold-induced endothelial injury described above.

### Histology and immunohistochemistry

Forty-eight hours after triggering, all rabbits were killed by an overdose of intravenous pentobarbital sodium. The right carotid arteries were perfused with phosphate-buffered saline, and artery specimens (3 cm in length) were quickly removed from the right carotid arteries. Each specimen was then cut into 2 pieces. One piece was fixed in 10% buffered formalin and embedded in paraffin for light microscopy and immunohistochemistry, while the other piece was fixed in 3% glutaraldehyde and postfixed in 1% osmium tetroxide, then processed routinely for transmission electron microscopy (JEM-1230, Japan). To characterize the general architecture of the atherosclerotic plaques, serial 4-μm-thick cross sections were cut and mounted on glass slides, and then stained with hematoxylin and eosin (H&E). Cellular composition, cell proliferation, cell apoptosis, and the presence of tissue factor were characterized immunocytochemically. The primary monoclonal antibodies used were as follows: an anti-smooth muscle α-actin antibody (Kangwei) to identify SMCs, anti-CD68 antibody (Kangwei) to identify macrophages, anti-factor VIII antibody (Maixin) to identify endothelial cells, anti-CD45RO antibody (Maixin) to identify T cells, anti-proliferating cell nuclear antigen (PCNA) antibody (Maixin) to detect proliferating cells, and anti-p53 antibody (Kangwei)to identify apoptotic cells.

### Biochemical analysis

Total cholesterol (TC), triglyceride(TG), and low-density lipoprotein(LDL) measurements were obtained by enzymatic assays of blood samples collected from the rabbits 8 weeks after the high-fat diet. The expression of high-sensitivity C-reactive protein (hs-CRP) was determined by enzyme-linked immunosorbent assay of blood samples collected from the rabbits before triggering and 48 hours after triggering. This was done with the ADL Diagnostic Kit (ADL, USA) procedure for hs-CRP. Plasma fibrinogen was determined by the Clauss method, and platelet count was determined by a Coulter counter from blood samples obtained before triggering and 48 hours after triggering.

### Statistical analysis

Diet-induced changes in the lipid levels were compared by paired Student's t test, as were hematological changes before and after attempted triggering. Student-Newman-Keul's test was used to assess statistical significance between the two groups. We considered differences to be significant at P < 0.05. All values were expressed as means ± SD.

## Results

### Extent of atherosclerotic plaque and plaque rupture/thrombosis

**Figure 1:**
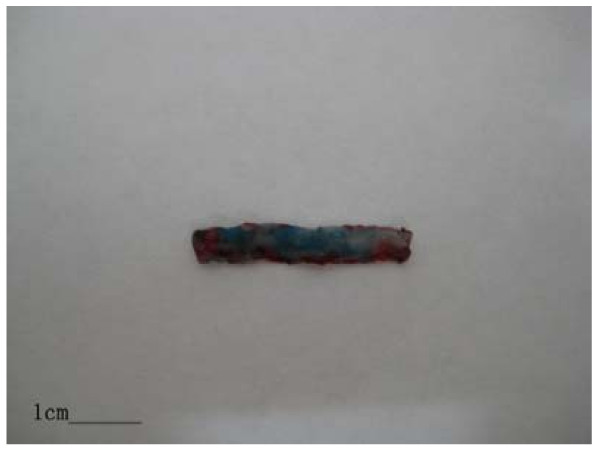
**Evans blue staining**. The right carotid artery injured by liquid nitrogen become dark blue (bar represents 1 cm).

All rabbits survived throughout the duration of the experiment. Evans blue staining showed that the intimal surface of right carotid artery injured by liquid nitrogen became dark blue (Fig. 1), but the left carotid artery had a normal color.

Eight weeks after cold-induced endothelial injury, each of the 3 rabbits that were taken to observe the extent of atherosclerotic plaques in group B showed extensive sheets of elevated white-yellow plaques, but had no spontaneous plaque rupture or thrombosis. At 48 hours after triggering, all of the remaining rabbits in group B had suffered plaque rupture or showed occlusive thrombus formation, while the intima of the right carotid arteries in group A appeared normal by gross inspection, and no atherosclerotic plaque or thrombi were noted.

### Histologic and immunohistochemical features of cold-induced plaques

**Figure 2:**
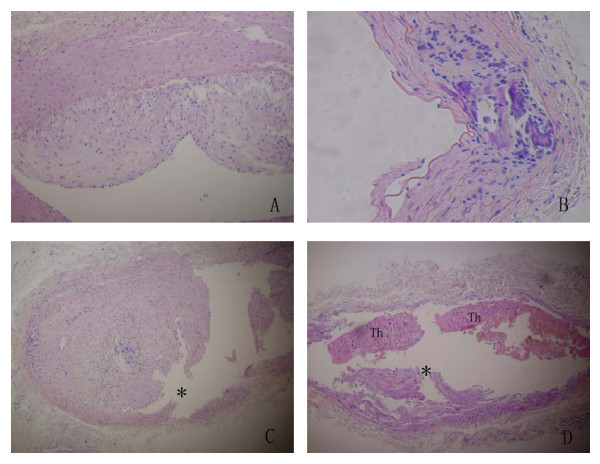
**Atherosclerotic plaque and plaque rupture/thrombosis**. (A) Stable plaque with an infiltration predominantly composed of foam cells. (B)Vulnerable plaque with thin fibrous cap, necrotic lipid core, many inflammatory cell infiltration and heavy calcification.(C)Eccentric plaque with plaque rupture at the cap and the shoulder region(asterisk).(D)Plaque rupture(asterisk)with occlusive thrombi(Th)(all H & E, A, original magnification, 20 ×; B, original magnification, 40 ×; C and D, original magnification, 10 ×).

Light microscopy of arterial samples from group A showed normal vascular histology. The samples from group B had extensive plaques composed of lipid-containing macrophages (foam cells), extracellular lipid collections, fibrous tissue, and calcification (Fig. 2, Panels A and B).

Arterial samples of disrupted plaques from group B showed that the plaques were usually broken at the caps or at the shoulder regions (Fig. 2, Panel C). Light microscopic examination of adjacent serial sections from disrupted plaques revealed cylindrical and round-edged white thrombi that were firmly attached to the arterial wall, red clots were loosely attached to the ends of the white thrombi, and early organization and inflammatory cell infiltration were present within the thrombi. (Fig. 2, Panel D).

**Figure 3:**
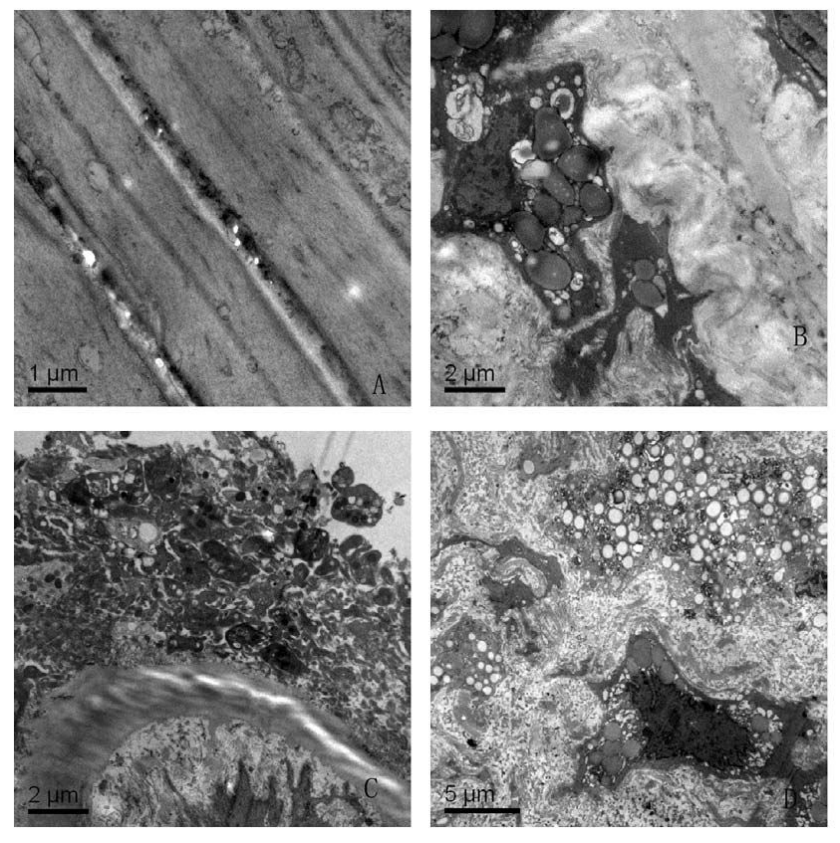
**Transmission electron micrograph of right carotid artery specimens**. (A)The smooth muscle cells in the media are spindle-shaped and well-arranged (bar represents 1 μm, magnification × 20 000). (B) The internal elastic lamina is loose. Many lipid droplets deposit in the intima and media.Furthermore, SMCs have died by disintegration into myriad vesicles (bar represents 1 μm, magnification × 10 000). (C)The intima is irregularly thickened and multilaminated, the internal elastic lamina is thickened, and the SMCs grow vertically to the internal elastic lamina(bar represents 2 μm, magnification × 10 000). (D) The smooth muscle cells in the media are disordered and filled with small cell bodies and lipid droplets, the collagen fibrils significantly increase, and the apoptotic bodies, pycnosis of the nuclei could be found(bar represents 5 μm, magnification × 5000).

Electron micrographs showed that the intimal surfaces of arteries from noninjured rabbits in group A were smooth and clean, the smooth muscle cells (SMCs) were well-arranged, and no lipid droplets were noted(Fig. 3, Panel A). However, transmission electron microscopy of atherosclerotic plaques from rabbits in group B indicated that smooth muscle cells were crowded with lipid droplets and smaller cell bodies. The basal laminae around the SMCs were irregularly thickened and multilaminated. The internal elastic lamina remained intact, but was thickened and denatured. The collagen fibrils had significantly increased in the media, and a large number of lipids had infiltrated into the thickened intima (Fig. 3, PanelB D).

**Figure 4:**
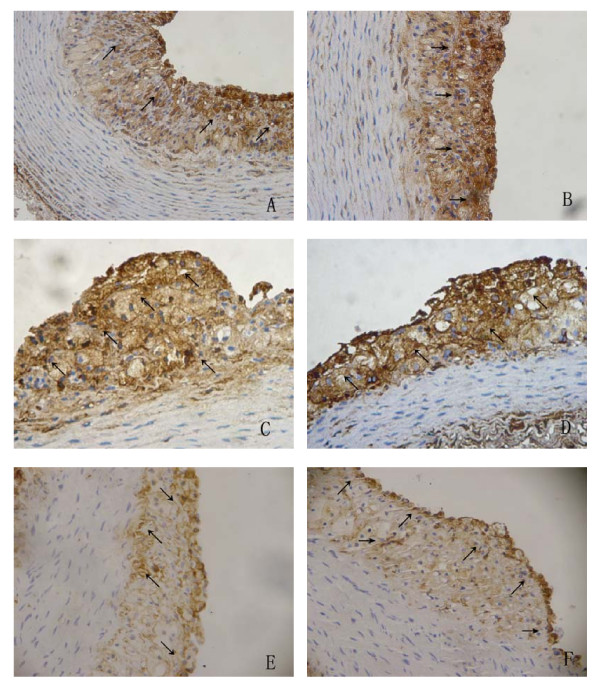
**Immunohistochemistry stains of atherosclerotic plaques.** (A) a-actin–positive SMCs (arrow). (B) CD68-positive macrophages(arrow). (C) CD45RO-positive T cells (arrow). (D) Factor VIII-positive endothelial cells (arrow). (E) p53-positive apoptotic cells (arrow). (F) PCNA-positive proliferating cells (arrow) (all DAB,A-F,original magnification, 20 x).

Results of immunohistochemistry showed that these atherosclerotic plaques contained CD68-positive macrophages, CD45RO-positive T cells, α-actin-positive SMCs, factor VIII-positive endothelial cells, PCNA-positive proliferating cells, and p53-positive apoptotic cells (Fig. 4).

### Serum lipid levels

Prior to the high-fat diet, the baseline of serum lipid levels (TC, LDL, and TG) did not differ between the two groups. However, 8 weeks after the high-fat diet, the serum lipid levels were significantly higher than baseline, but there were no significant differences between the two groups (Table 1).

**Table 1:** Lipid levels of the two groups (mmol/L).

	before high-fat diet			8 weeks after high-fat diet		
	
Group	TC	LDL	TG	TC	LDL	TG
Group A	2.50 ± 0.45	1.05 ± 0.38	1.08 ± 0.47	30.76 ± 5.58^a^	22.72 ± 6.61^a^	4.36 ± 1.63^b^

Group B	2.43 ± 0.54	1.10 ± 0.42	1.15 ± 0.39	31.88 ± 3.83^a^	23.69 ± 5.02^a^	4.58 ± 1.57^b^

Note: a:*P *< 0.001;b:*P *< 0.01, compared with baseline before high-fat diet

Hematological changes after triggering There were no significant differences between the two groups before triggering. However, 48 hour after triggering, the levels of hs-CRP, platelet counts and plasma fibrinogen in group B were significantly higher than the levels observed before triggering. The levels of hs-CRP, platelet counts, and plasma fibrinogen in group A did not increase significantly after triggering (Table 2).

**Table 2:** Hematological changes after triggering.

	hs-CRP(mg/L)		Platelet counts(/mm^3^)		Plasma fibrinogen(mg/dL)	
	
Group	before triggering	after triggering	before triggering	after triggering	before triggering	after triggering
Group A	0.96 ± 0.35	1.05 ± 0.24	212.6 ± 114.2 × 10^3^	220.3 ± 105.6 × 10^3^	206.8 ± 53.5	215.2 ± 46.4

Group B	0.92 ± 0.28	3.84 ± 0.73^a^	218.3 ± 97.0 × 10^3^	953.6 ± 307.0 × 10^3b^	213.7 ± 49.1	536.3 ± 78.6^b^

Note: a:*P *< 0.01; b: *P *< 0.001, compared with the levels before triggering.

## Discussion

### Features of cold-induced plaques

We successfully developed a novel animal model of atherosclerotic plaque that is associated with true plaque rupture and also with the rupture-driven platelet-and fibrin-rich thrombus formation caused by cold-induced endothelial injury in high-fat-fed rabbits.

Our model has clear characteristics of human atherosclerotic plaques as we observed lipid-containing macrophages (foam cells), T cells, extracellular lipid collections, a fibrous plaque cap, and calcification. The vulnerable or disrupted plaques were histologically characterized by a necrotic lipid core, a thin fibrous cap with inflammatory cells, and cylindrical and round-edged white occlusive thrombi adhering to the arterial wall. Moreover, cell proliferation, cell apoptosis, and the presence of tissue factor were noted in the plaques, and the serum lipid levels were significantly higher than baseline 8 weeks after the high-fat diet. More importantly, the expression of hs-CRP, platelet counts, and plasma fibrinogen were significantly higher in the rabbits with ruptured plaques. All of these features indicated that the plaques we induced are similar to those observed in patients with coronary heart disease and stroke [1,14,15].

### Comparison with other models

At present, there is no gold standard animal model for plaque rupture and thrombosis. One of the major drawbacks of the existing models is the lack of an end-stage atherosclerosis that shows plaque rupture and platelet-and fibrin-rich thrombi. This is a very important limitation because myocardial infarction or cerebral infarction in humans is not caused by plaque rupture *per se*, but by the formation of the platelet-and fibrin-rich occlusive thrombi [13]. In addition, the balloon-induced and biological or mechanical triggering rabbit models [3-6] are very labor-intensive and expensive, and the animals frequently require over eight months to develop significant lesions. Although pharmacological triggering models have been shown to develop acute aortic thrombi, these thrombi are primarily associated with endothelial toxicity and do not represent true plaque rupture. Some plaques of the WHHL model rabbit contain a lipid core and a thin fibrous cap similar to human vulnerable plaques, but true plaque rupture and occlusive thrombus are not observed in WHHL rabbits [7,16]. In mouse studies [8-12], the period of onset is too long and the rate of plaque rupture and luminal thrombi is too low to observe any effects of intervention. Furthermore, spontaneous plaque rupture in mice is merely an intraplaque hemorrhage and not a true plaque rupture. As well, the thrombi are mostly not organized and nonocclusive. In humans, the plaque rupture and occlusive thrombus formation that actually kill and disable humans are linked, yet either one can occur without the other.

To the best of our knowledge, our model is the first report of an animal model that demonstrates direct evidence of plaque rupture and the rupture-driven platelet-and fibrin-rich occlusive thrombus formation that is similar to its human counterpart. The other distinguishing advantages of our model over other models are that it is simpler, has a shorter duration, is less expensive, and is more reproducible, as our experimental data indicated. Because atherosclerotic plaque and plaque rupture/thrombosis occurred in all cold-injured rabbits, and none of the rabbits died during the experiments, this model also provides both a lower mortality and a higher yield of triggering. These advantages will greatly facilitate ensuing study of human-type plaque rupture on a large scale.

### Mechanisms of the cold-induced model

Atherothrombosis is a complex disease that includes both atherosclerosis and thrombosis. Over the years, it has been recognized that it is plaque composition, rather than plaque size or stenosis severity, that is important for plaque rupture and subsequent thrombosis. The underlying mechanisms of atherothrombosis include endothelial dysfunction, lipid accumulation, and enhanced inflammatory involvement, which result in plaque disruption and subsequent thrombosis [17]. Analyses of human plaques have demonstrated that disrupted plaques have significantly less collagen, a low number of smooth muscle cells, and a high inflammatory cell content [18,19]. Plaque rupture occurs as a result of interactions between extrinsic triggering factors and the intrinsic vulnerability of the plaque, when forces acting on the plaque exceed its tensile strength [20-22]. Plaques with a large necrotic lipid core, increased inflammatory cell infiltration, and a thin fibrous cap appear to be particularly vulnerable to rupture [23]. One important issue in the prediction of vulnerability of a plaque to rupture is having the ability to determine the mechanical stress in the wall of the pathological artery and, more specifically, in the fibrous cap. The currently favored hypothesis is that plaque rupture in the fibrous cap initiates thrombus formation by exposing blood either to collagen in the extracellular matrix or to previously sequestered tissue factor associated with lipid-laden macrophages, or to both. Fresh occlusion is identified by a luminal thrombus containing platelet aggregates interspersed with inflammatory cells, and a paucity of red blood cells.

It is well known that endothelial injury is a key event in the pathogenesis of atherosclerosis. Our experimental approach was based on the hypothesis that an atherosclerotic plaque can be initiated by cold-induced endothelial injury with liquid nitrogen, and that the plaque can be ruptured at will by later triggering with liquid nitrogen. This hypothesis is supported by the finding that when endothelium is frozen and thawed immediately, various ultrastructural alterations occur. For example, membranous structures are extensively damaged and endothelial cell apoptosis or death occurs through intra- and extracellular ice crystal formation [24,25]. Along with the destruction of barrier function, lipoproteins enter the vessel wall, promoting the recruitment of monocytes, which in turn imbibe lipids and become foam cells, and atherosclerotic plaques can then develop [26]. When the plaques are triggered by liquid nitrogen, the apoptotic rate of endothelial cells and smooth muscle cells increases and the proportion of collagen production decreases at the position of the plaque. The triggering action then forces the plaque contents through the thin fibrous cap or weakened shoulder region, producing an effect like a volcanic eruption[27]. Thus, vulnerable plaques were grossly disrupted as a result of the local increase in stress caused by cold triggering, which is very similar to ACS triggered by acute events. The circulating platelets are recruited to the site of injury, where they become a major component of the developing thrombus. Platelet thrombus formation and fibrin deposition occur concomitantly, and the occlusive thrombi then lead to acute ischemic events.

In other words, the cold-induced lesion in our model is reminiscent of human plaque rupture in terms of endothelial injury, lipid deposition, macrophage infiltration, aggregation of platelets, a relatively hypercoagulable state, and a triggering as a result of activity such as local vasospasm.

### Potential usefulness of the cold-induced model

Because atherosclerotic plaque rupture occurs in a random fashion, by its very nature it is difficult to study directly in humans. The merits of our model make it more feasible to evaluate treatment strategies designed to stabilize vulnerable plaques (primary prevention), to diminish thrombosis after disruption, and to promote the curative effect of ruptured plaques (secondary prevention). In addition, the model also can help us to develop new drugs or other therapies that are able to prevent plaque rupture and thrombosis from happening on a large scale. Finally, this model can help us to identify biomarkers and also to accurately image vulnerable plaques or ruptured plaques.

In this study, we demonstrated a rabbit model of human plaque rupture that shows direct evidence for plaque rupture and rupture-driven platelet-and fibrin-rich occlusive thrombus formation for the first time. The model is simple, fast, inexpensive, and reproducible, and has a low mortality and a high yield of triggering. We hope that this model will help us to understand the mechanism of human plaque rupture and also to reduce the incidence of thrombus-induced heart attack and stroke.

## Competing interests

The authors declare that they have no competing interests.

## Authors' contributions

S-MF conceived of the study, designed the experiment, carried out the main experiment and drafted the manuscript. Q-HZ participated in its design and coordination. Z-XJ carried out the biochemical analysis and helped to perform the statistical analysis. All authors read and approved the final manuscript.
